# The Apostolic Succession

**Published:** 1870-09

**Authors:** 


### Editorial.

***The Apostolic Succession.***

At the recent semi-annual meeting of the Minnesota State Med. Society, Dr. Solomon Blood expressed his regret

"That Dr. Stone chose the name which he did for the new journal. *The Chicago Medical Examiner* was first published under the same name, and I now have in my possession eight or ten volumes of the 'Northwestern Medical and Surgical Journal.' I have made hundreds of pages of references to the 'N. W. M. & S. Journal' in my note book, and wish that the name of this one might be changed."

We cordially agree with Dr. Blood in the desire that the name of our young but promising contemporary should be changed, for the same reasons which prompted our predecessors in the editorial conduct of this Journal to drop the cumbersome and inappropriate prefix Northwestern. St. Paul is no more, relatively, in the Northwest than Chicago was fifteen years since. The *Oregon Reporter* would have done better to take this designation than the one now used by several of its contemporaries, and it is in the Northwest. The prefix "Surgical" is obviously unnecessary. No one speaks of the Medical and Surgical profession, for the term Medical strictly embraces both, and the second prefix is mere tautology. But this is a matter of taste which, with the Minnesota Society, we refer to Dr. Stone—suggesting to him, however, that his contemporaries who will, of course, extract largely from his pages, would be pleased at relief from writing out the "Alexandrine" at the end of each of their scissorings. Just imagine the discomfort involved in quoting a passage from our Boston contemporary: "*The Journal of the Gynæcological Society of Boston.*" We positively omit many capital scissorings from that organ of the woman specialty, merely to avoid the manual labor of writing out its sesquipedalian title, and then invariably correcting the printer's proof which puts *œ* instead of *æ* every time. "What's in a name?" A great deal too much, very often.

But just now our object is to correct the misapprehension into which Dr. Blood has fallen. "*The Chicago Medical Examiner*"

was never published under the name "Northwestern Medical and Surgical Journal," but was started *ab initio* in 1859. It is not in the *succession*. This Journal is the legitimate and only successor and representative of the old "*Northwestern*," etc. etc. It is now in its twenty-seventh year, and, we are happy to say, in the most prosperous condition it has ever known. The present writer expects to continue in its editorial chair for the next quarter of a century of its career, or, at least, as long as it may be in his power to assist the cheerful monthly mentioned by Dr. Blood, in its appointed work of "elevating the profession" —by "blowing it up."

We have upon our shelves a complete file of the "Illinois M. & S. Journal," then the "Northwestern M. & S. J.," and since then the plain *Chicago Medical Journal*. We have its old mail books and ledgers, and, alas, its thousands of dollars of uncollected subscriptions, which our present publishers, fortunately for themselves, refused to have anything to do with, preferring the exclusively "cash-in-advance" system for the future. We will sell out the arrearages to any ambitious medical gentleman who wishes to start a new medical journal here or elsewhere.

